# Desflurane and remifentanil anesthesia in a child with citrin deficiency: A case report

**DOI:** 10.1097/MD.0000000000028954

**Published:** 2022-03-04

**Authors:** Kanghui Kim, Sung Mee Jung

**Affiliations:** 1 Department of Anesthesiology and Pain Medicine, Yeungnam University College of Medicine, Daegu, Republic of Korea.

**Keywords:** citrin, citrullinemia, desflurane, general anesthesia, hyperammonemia, remifentanil

## Abstract

**Rationale:**

Hyperammonemia, metabolic derangement, and/or the prolonged effects of anesthetics may lead to delayed emergence from general anesthesia as well as the onset of type 2 citrullinemia, even in compensated patients with citrin deficiency.

**Patient concern:**

A 5-year-old girl with citrin deficiency was scheduled for blepharoplasty under general anesthesia. She developed hyperammonemia with temporary interruption of medication for a few days before surgery.

**Diagnosis:**

The patient was genetically diagnosed as citrin deficiency with a mutation in the *SLC25A13* gene via newborn screening for metabolic disorders. Her citrulline and ammonia levels were well-controlled with arginine medication and protein-rich diet. Her elevated ammonia level by temporary interruption of medication was corrected with resumption of arginine medication and protein-rich diet before surgery.

**Interventions:**

We used desflurane and remifentanil for general anesthesia to avoid hyperammonemia and delayed emergence. End-tidal desflurane concentration and anesthetic depth were carefully monitored to avoid excessive anesthesia.

**Outcomes:**

She recovered consciousness with slightly increased ammonia level immediately after anesthesia.

**Lessions:**

General anesthesia of the shortest duration with the least metabolized drugs using desflurane and remifentanil, would be beneficial for rapid emergence in surgical patients with citrin deficiency. Maintenance of nitrogen scavenging medication, a protein-rich diet, and serial measurement of ammonia levels in the perioperative period are also important for avoiding hyperammonemia-related neurological dysfunction.

## 1 Introduction

Citrin, an aspartate glutamate carrier in the mitochondrial inner membrane, plays a role in the supply of aspartate from the mitochondria to the cytosol and malate aspartate nicotinamide adenine dinucleotide hydrogen shuttle.^[1]^ Citrin deficiency is an autosomal recessive disorder caused by mutations in the *SLC25A13* gene on chromosome 7q21.3. Lack of aspartate supply from the mitochondria to the cytosol impairs gluconeogenesis, glycolysis, and fatty acid oxidation in the liver. In addition, the lack of aspartate required for the argininosuccinate synthase reaction in urea synthesis and impaired glutamine synthase function for glutamine synthesis from glutamate and ammonia in hepatocytes causes accumulation of citrulline and ammonia in the body.

Clinically, citrin deficiency has 3 age-dependent phenotypes: neonatal intrahepatic cholestasis caused by citrin deficiency (NICCD, Online Mendelian Inheritance in Man #605814), adaptation/compensation stage with unique food preference during childhood, and adult-onset type 2 citrullinemia (CTLN2, Online Mendelian Inheritance in Man #603471). The clinical features in most infants with NICCD spontaneously resolve between 6 months and 1 year of life with a protein-rich and carbohydrate-poor diet, despite the rare occurrence of prolonged cholestasis and irreversible hepatic failure.^[2]^ Some infant patients may not present with any NICCD-related clinical manifestations and are genetically diagnosed with citrin deficiency following identification via a newborn screening test. Only a few patients with citrin deficiency develop CTLN2, which is characterized by serious and recurrent hyperammonemia, liver steatosis, and neuropsychiatric symptoms such as sudden aberrant behavior and disturbance of consciousness after >10 years.^[3]^ It is triggered by alcohol intake, excessive carbohydrate ingestion, sepsis, fasting, medication, or surgery,^[4]^ and has a poor prognosis without liver transplantation despite intensive treatment. Between NICCD and the onset of CTLN2, most patients with citrin deficiency enter a compensation (asymptomatic) stage during childhood. However, even during the asymptomatic period, affected children can have metabolic abnormalities, such as sustained citrullinemia, hypercholesterolemia, and augmented oxidative stress.^[5]^

The effect of anesthetic techniques and regimens on metabolic changes, especially ammonia levels, should be carefully considered to prevent the onset of CTLN2 and achieve rapid recovery in the perioperative period. Previous case studies reported delayed emergence from general anesthesia in a child with citrullinemia^[6]^ and spinal anesthesia without significant neurologic events despite recurrent hyperammonemic episodes in adult patients with CTLN2.^[7]^ However, case reports of anesthetic management in patients with citrin deficiency at different clinical stages are still sparse. Here, we report the case of a 5-year-old girl with citrin deficiency who recovered consciousness immediately after general anesthesia using desflurane and remifentanil for eye surgery. In addition, we considered the findings of previous studies.

## 2 Case report

This case study was approved by institutional review board (YUH-2021-08-066) and obtained a written informed consent from parent for its publication. A 5-year-old girl (17.3 kg, 110.8 cm, body mass index = 14.1 kg/m^2^) with citrin deficiency was admitted for elective bilateral blepharoplasty under general anesthesia. Her family history was unremarkable. She was identified via newborn screening for metabolic disorders without clinical symptoms and was genetically diagnosed with citrin deficiency with compound heterozygotes of c.674C>CA; p.[Ser225Ter] in Exon7; and lVS16ins3 kb in intron 16 of the *SLC25A13* gene on chromosome 7q21.3.

**Figure 1:**
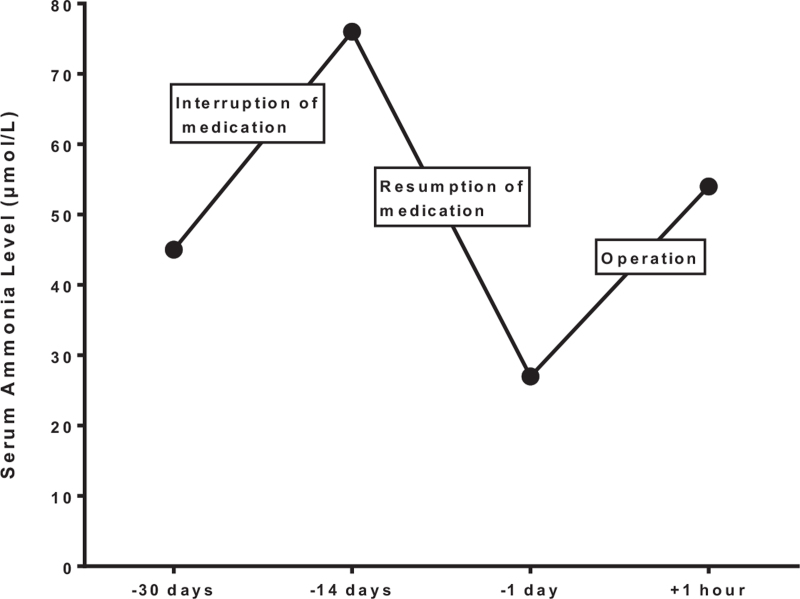
Change of serum ammonia levels in the perioperative period.

Her blood and urine citrulline and ammonia levels, liver function, and electrolyte levels measured at 6-month intervals were well controlled with L-arginine supplementation 3 times per day and high protein and low carbohydrate diet (Fig. 1). However, transient interruption of medication for 2 weeks before surgery resulted in an abrupt increase in serum ammonia (76 μmol/L; reference level 11–35 μmol/L) and cholesterol levels (254 mg/dL; reference level 120–200 mg/dL). After resumption of medication for 1 week, her ammonia level decreased to 27 μmol/L.

On admission for surgery, she had no clinical symptoms or signs of citrin deficiency. The operation was planned for early in the morning to minimize the fasting time. She received arginine 250 mg without premedication and was transferred to the operating room with her mother to reduce separation anxiety. On arrival in the operating room, the patient was monitored using a pulse oximeter, electrocardiogram, non-invasive blood pressure, and bispectral index (BIS). General anesthesia was induced using intravenous administration of propofol (20 mg) premixed with lidocaine 20 mg with infusion of remifentanil (0.05 μg/kg/min). Endotracheal intubation was facilitated after administration of rocuronium (0.5 mg/kg). Anesthesia was maintained by inhalation of desflurane with 50% oxygen and infusion of remifentanil (0.025 μg/kg/min) during surgery. Intraoperative fluid was administered at a rate of 60 mL/h with 5% dextrose plus NAK2 (0.45% NaCl and 0.15% KCl) solution. The BIS scores with end-tidal desflurane concentrations between 5% and 6% were maintained between 40 and 60 during anesthesia. Her temperature was maintained at 36.8 to 37.1 °C and the blood glucose level at the end of surgery was 96 mg/dL.

The surgery and general anesthesia concluded after 55 minutes. Upon completion of the operation, spontaneous respiration was immediately restored with administration of pyridostigmine (0.2 mg/kg) and glycopyrrolate (0.008 mg/kg). She opened her eyes on verbal commands, and extubation was successfully performed without complications 5 minutes after discontinuation of desflurane and remifentanil (BIS score of 95 and end-tidal desflurane concentration of 0.7%). She had neither neurologic symptoms nor complaints of pain during her stay in post-anesthetic care unit. The serum ammonia level 1 hour after surgery increased to 54 μmol/L (Fig. 1). The patient was discharged without any complications on the first postoperative day.

## 3 Discussion

Citrin deficiency was previously thought to be a rare hereditary disease restricted to the Japanese population but is now considered a pan-ethnic disease that is prevalent in East Asia.^[8,9]^ The calculated frequency of homozygotes from the carrier rates (1/112) of mutations in *SLC25A13* among Koreans is 1/50,000,^[9]^ suggesting the likelihood of hospitals encountering surgical patients with citrin deficiency in Korea. The impairment of glycolysis, lipogenesis, ATP synthesis, and ammonia detoxification systems in patients with citrin deficiency can lead to metabolic deterioration and hyperammonemia in response to catabolic surgical stress and result in an unexpected response to anesthetic drugs and/or prolonged recovery of consciousness after general anesthesia.^[6]^ Although blood ammonia levels are not always consistent with the clinical manifestations,^[4,7]^ the total duration of hyperammonemic coma and maximum ammonia level negatively correlate with the patient's neurological outcome, especially in the developing brain.^[10]^ Therefore, previous studies have emphasized anesthetic techniques and regimens to minimize metabolic burden on the liver and reduce stress response to surgery and meticulous management of perioperative ammonia levels.^[6,7,11]^

Despite her low BMI, our patient remained clinically asymptomatic and maintained ammonia and amino acid levels within the reference range with L-arginine medication and diet therapy. However, an abrupt increase in ammonia level after temporary interruption of nitrogen scavenging medication suggested that she was likely to develop metabolic deterioration and hyperammonemia in response to surgical stress in the perioperative period. We optimized blood ammonia levels by maintaining arginine medication with monitoring serum ammonia levels before and after surgery. In children with citrin deficiency, hypoglycemia is frequently noted during caloric deprivation because of the preference for a low-carbohydrate diet, suppression of gluconeogenesis, and decreased hepatic glycogen storage due to energy deficit.^[12]^ In contrast, persistent hyperglycemia is prone to increase glucose uptake and accelerate metabolite accumulation, ATP depletion, and hepatocyte damage. Thus, we maintained normoglycemia during the perioperative period by minimizing fasting time while administering intravenous fluids with low glucose (5% dextrose) in our patient.

Delayed awakening after anesthesia remains a concern in surgical patients with citrullinemia. Previous case studies chose regional anesthesia as possible for early detection of hyperammonemia-related conscious disturbance and avoiding metabolic burden in surgical patients with citrullinemia.^[7]^ We focused on selecting the least metabolizing and short-acting drugs to prevent hyperammonemia, disturbance of blood glucose control, and residual effect of anesthetic agents in our patient requiring general anesthesia. Desflurane and remifentanil used for maintenance of anesthesia and analgesia enabled rapid recovery of consciousness after surgery, with a slight increase in postoperative ammonia level (54 μmol/L). No study has investigated the effect of desflurane maintenance on serum ammonia levels in humans. However, considering that desflurane possesses the lowest solubility in blood and other body tissues, the lowest metabolism (0.02%) of halogenated anesthetics, and does not require metabolism for elimination following cessation of administration,^[13]^ it is probably suitable as the main anesthetic agent for patients with impaired nitrogen metabolism. Remifentanil also provides the advantage of speedy recovery owing to its shortest context-sensitive half-life. Although the glycine contained in remifentanil is metabolized to ammonia, blood ammonia levels remain within the normal range, even after long-term infusion of remifentanil.^[14]^

In contrast, a previous case study reported delayed awakening and sustained discoordination and ataxia despite recovery of spontaneous ventilation and slightly increased ammonia levels (52.3 μmol/L) after sevoflurane anesthesia for dental procedure in a boy with citrullinemia.^[6]^ Although adjuvants, such as morphine, for analgesia may contribute to delayed emergence, the authors concluded naloxone treatment for opioid reversal did not improve the patient's consciousness in their case. Therefore, the prolonged effect of anesthetic agents should be considered as one of the main reasons for delayed emergence in surgical patients with citrullinemia. They used sevoflurane anesthesia for 4 hours, while we used desflurane anesthesia for approximately 1 hour.

The elimination of inhaled anesthetics after discontinuation is determined by ventilation of the lungs and the pharmacokinetics of the anesthetic agent. The rapid emergence after desflurane anesthesia in our patients may be explained by pharmacokinetic characteristics rather than ventilation due to spontaneous ventilation with complete reversal of neuromuscular blockade in both cases. For recovery of consciousness, the required decrease in inhaled anesthetic concentration would be nearly 80% and even greater in the presence of any other hypnotics and/or analgesics.^[15]^ The difference in decrement times for inhaled anesthetics roughly parallels the blood/gas solubility and increases with increasing duration of anesthesia. The 90% decrement time for awakening and orientation is shorter after desflurane (blood/gas solubility approximately 30% less than that of sevoflurane) regardless of the duration of anesthesia, but substantially longer after sevoflurane anesthesia after a long period of anesthesia.^[15]^ Therefore, desflurane, which has both lower blood/gas solubility and tissue deposition, has faster washout from the brain and lungs after discontinuation of administration and is probably more suitable than sevoflurane for patients with metabolic disease, especially in procedures of long duration. However, it is still unclear how genetic defects in the metabolic pathway influence the response to and duration of anesthesia in surgical patients with citrin deficiency. It would be helpful to carefully titrate anesthetic dosage guided by the end-tidal concentration and anesthetic depth monitoring to avoid anesthetic overdose associated with a metabolic deficit resulting in delayed emergence.

Although our patient was free of pain, postoperative pain relief is important to avoid onset of CTLN2 triggered by surgical stress in patients with citrin deficiency. Choice of analgesic and its dosing is challenging owing to a lack of evidence-based guidelines for the use of analgesics in these patients. Because liver plays a predominant role in the metabolism and elimination of the majority of analgesics including acetaminophen, nonsteroidal anti-inflammatory drugs, and opioids, the severity of hepatic impairment in patients with citrin deficiency should be carefully evaluated. Considering that acetaminophen may trigger onset of CTLN2,^[16]^ we suggested the use of ketorolac without hepatotoxic effect to relieve mild to moderate pain.^[17]^ For relief of moderate to severe pain, short-term use of phenylpiperidine opioids such as fentanyl at reduced doses is more likely to avoid accumulation and adverse events than morphine.^[6]^ The use of opioid in patients with citrin deficiency should be carefully titrated in order to minimize dose-dependent side effects and monitored for any sign of hepatic encephalopathy such as disturbance of consciousness.

In summary, desflurane and remifentanil, which has a short duration and very low metabolism, may provide rapid emergence from general anesthesia in patients with citrin deficiency. The administration of inhaled anesthetics should be guided by monitoring end-tidal concentration and anesthetic depth to avoid overdose associated with metabolic deficits. Perioperative maintenance of nitrogen scavenging medication and a protein-rich and low-carbohydrate diet in patients with citrin deficiency is also important to prevent hyperammonemia in response to surgical stress, even in the compensation period.

## Author contributions

**Conceptualization:** Kanghui Kim, Sung Mee Jung.

**Data curation:** Kanghui Kim.

**Investigation:** Kanghui Kim.

**Supervision:** Sung Mee Jung.

**Validation:** Kanghui Kim.

**Writing – original draft:** Kanghui Kim, Sung Mee Jung.

**Writing – review & editing:** Kanghui Kim, Sung Mee Jung.
